# Can Non‐Neurosurgeons Operate on Traumatic Brain Injuries in Non‐Metropolitan Areas? A Scoping Review

**DOI:** 10.1111/1742-6723.70055

**Published:** 2025-05-21

**Authors:** Lauren Bosley, Clinton Gibbs, Eunah Joo, Geoffrey Dobson

**Affiliations:** ^1^ College of Medicine and Dentistry James Cook University Townsville Australia; ^2^ Retrieval Services Queensland Queensland Health Townsville Australia; ^3^ Emergency Department Townsville University Hospital, Queensland Health Townsville Australia

**Keywords:** brain injuries, decompressive craniectomy, emergency medical services, hospitals, neurosurgery, rural, traumatic

## Abstract

Traumatic brain injuries (TBIs) with increased intracranial pressure (ICP) require time‐sensitive surgical intervention. In non‐metropolitan areas, neurosurgeons are often unavailable to provide definitive treatment. Therapeutic surgical intervention by a non‐neurosurgeon, for example, general surgeons, is a potential alternative; however, the feasibility and utility of non‐specialist intervention are poorly defined within the literature. A scoping review was conducted within Scopus, Emcare, MEDLINE and CINAHL for original literature about emergency neurosurgical interventions performed by a non‐neurosurgeon for TBIs in non‐metropolitan settings without prompt access to a neurosurgeon. This search yielded 20 studies that included over 2000 surgical interventions in 13 countries. General surgeons most commonly performed the procedures on patients with computed tomography (CT)‐confirmed lesions. Mortality rates were heterogeneous, ranging from 0% to 67% in small cohorts with variable follow‐up periods. Mortality was consistently higher in patients with subdural haematomas (SDHs) opposed to extradural haematomas (EDHs). Morbidity was measured in 13 studies, commonly via the Glasgow outcome scale (GOS). Most studies had access to remote neurosurgical advice via telehealth. Overall, these 20 studies provided incomplete information regarding mortality rates and functional outcomes from this alternative practise. The present study concludes that emergency decompression by a non‐neurosurgeon for patients with severe TBIs may be lifesaving for patients without timely access to a neurosurgical centre. Our study further highlights the need for further research, training and resource allocation, including strengthening telecommunication pathways, to support patient access to lifesaving neurosurgical interventions in these environments, and ultimately address surgical inequalities in rural and remote regions of the world.

## 1 Introduction

Traumatic brain injuries (TBIs) are a leading cause of morbidity and mortality worldwide, with increasing incidence. Non‐metropolitan populations are burdened by higher incidences and worse outcomes from TBIs relative to metropolitan populations, influenced by delays to accessing care and inadequate resources [1, 2]. A severe TBI is defined as a Glasgow coma scale (GCS) score of less than nine and includes patients with significant extradural haemorrhages (EDHs) and subdural haemorrhages (SDHs), for which surgical decompression is the recommended definitive management [3]. If surgical decompression occurs within 4 h of hospital presentation, studies have shown that mortality is significantly reduced [4, 5]. However, in non‐metropolitan areas, neurosurgeons are often not available within recommended timeframes [6]. Non‐metropolitan locations have variable definitions, with Australia identifying these regions by having a population of less than 100,000, restricted access to goods and services, and distance from a major city. Due to the smaller populations served, non‐metropolitan hospitals may not have specialist staff and infrastructure; thus, patients in need of this care are often transferred to larger hospitals [7]. The Neurosurgical Society of Australasia guidelines recommend that if patients are more than 2 h from a neurosurgical centre and have clinical or computed tomography (CT) signs of increased intracranial pressure (ICP), then non‐neurosurgeons should perform surgical decompression of TBIs [8]. Digital instructions and telehealth support can assist local doctors in performing these lifesaving procedures, with real‐time virtual support from neurosurgeons [6]. No systematic reviews to date are known to examine non‐neurosurgeons' performance of neurosurgical interventions for patients with increased ICP secondary to a TBI in these non‐metropolitan settings. Given the scarcity of data on this topic, this scoping review aims to evaluate the evidence of non‐neurosurgeons performing neurosurgical interventions for acute TBI management in non‐metropolitan settings that do not have a neurosurgical specialty. Identifying evidence of the success of these interventions by non‐neurosurgeons may highlight the need for and importance of providing non‐metropolitan centres and staff with the capability and resources to surgically decompress TBIs.

## 2 Method

### 2.1 Scoping Review

A scoping review registered with Open Science Framework (osf.io/4mepc) was conducted in accordance with the Preferred Reporting Items for Systematic Reviews and Meta‐Analyses (PRISMA) guidelines (Appendix S1) [9].

### 2.2 Search Strategy

**FIGURE 1:**
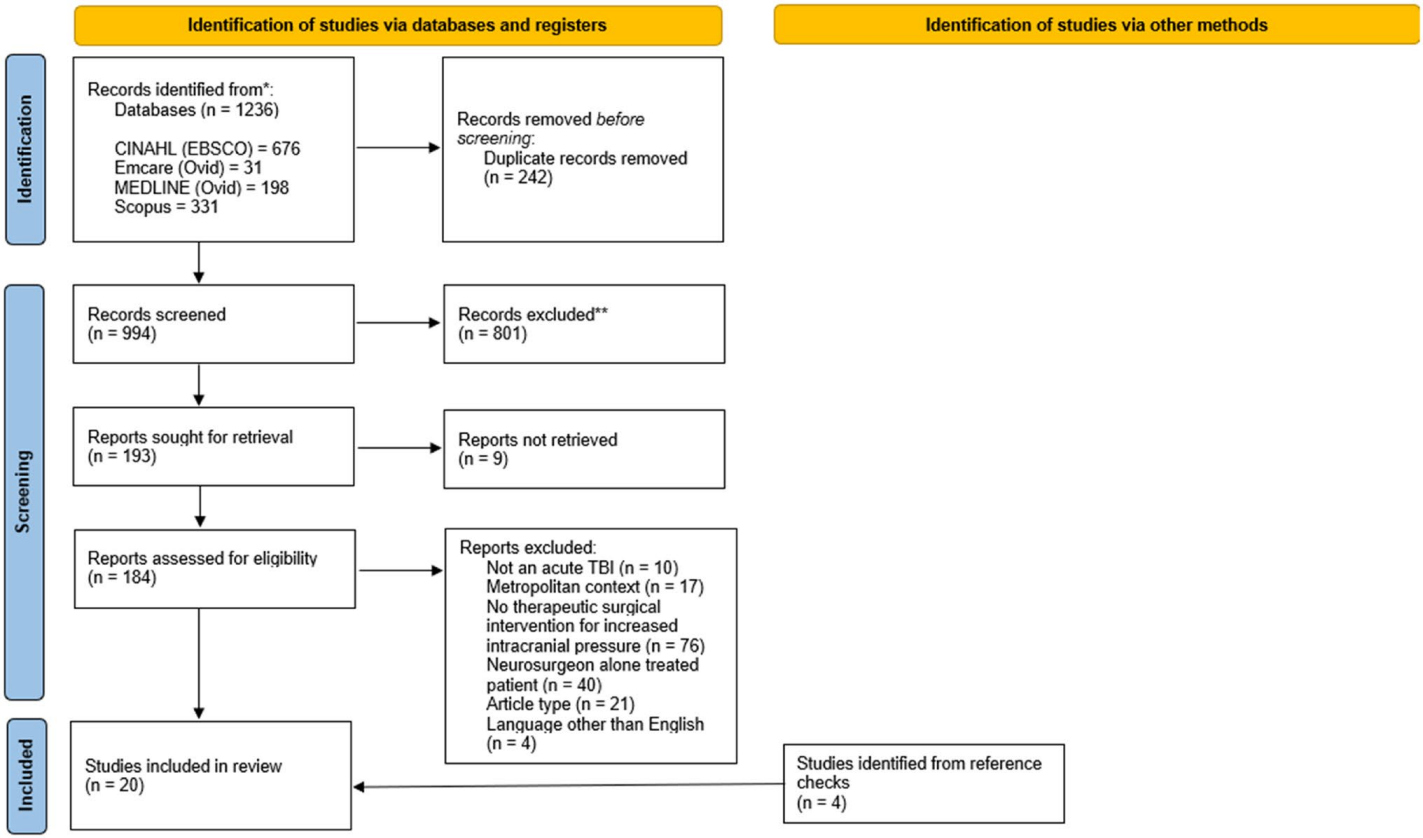
PRISMA flow diagram. PRISMA flow diagram of the study selection process. CINAHL, cumulative index of nursing and allied health; EBSCO, Elton B. Stephens Company.

All original research that explores therapeutic neurosurgical interventions by a non‐neurosurgeon for acute TBIs that was published in the searched databases was included. Literature searches were performed on Scopus, Emcare, MEDLINE and CINAHL for publications available to 31 May 2024, using subject headings and keywords relating to TBI (population), neurosurgical intervention (concept) and non‐metropolitan (context). Under the non‐metropolitan umbrella, this paper encapsulates regional, rural and remote hospitals without access to a neurosurgeon. The full subject headings and keywords used are reported in Appendix S2. Reference lists of all included studies were reviewed to identify four additional relevant studies. Search results were exported to a reference managing database (EndNote). Articles were excluded if they did not include all of the following criteria: a surgical treatment of acute TBI performed by a non‐neurosurgeon in a non‐metropolitan context. As per Figure 1, articles that were conference abstracts, reviews, letters to the editor, case studies, not in English and without full text availability were also excluded.

### 2.3 Study Selection

After removing duplicates, two investigators (L.B. and E.J.) independently screened titles and abstracts to identify eligible articles. Subsequently, full texts were reviewed by the same investigators, with a third investigator (C.G.) providing input on any inconsistencies in the screening.

### 2.4 Data Extraction

Data extracted included the dates, country, setting, population, study design, types of pathology treated, number of interventions performed, types of interventions if available, use of imaging, specialty of the operating clinician, mortality and other major findings related to patient outcomes.

### 2.5 Quality Assessment

Methodological quality was assessed using the Quality Assessment Tool for Studies with Diverse Designs (QATSDD) for non‐randomised studies with different designs [10]. Two authors (L.B. and E.J.) agreed on the grading of cumulative criteria for each article, with input from a third author (C.G.) as required. Results were totalled as a percentage of the maximum score to allow comparison across different methodologies. The result interpretation was that greater than 75% is considered high quality, 50%–75% good quality, 25%–50% moderate quality and less than 25% is poor quality [11].

## 3 Results

### 3.1 Study Characteristics and Quality Assessment

A total of 1236 potentially relevant records were retrieved from the search strategy, and after removal of 242 duplicates, 994 underwent title and abstract screening (Figure 1). Totally, 193 met inclusion for full text review, with nine articles unable to be retrieved. After applying the eligibility criteria, 16 articles were included for analysis. Reference checks of these articles identified four more relevant articles, resulting in 20 that underwent final analysis. Revision of the search strategy in response to these four additional articles was not performed, because broadening the search context to “neurosurgery” instead of specific neurosurgical procedures, or the population to exclude the “rural and remote” heading yielded many results that were not relevant to our question. Table 1 summarises the studies' characteristics. The included literature consisted of 17 case series and three surveys. The QATSDD scores reported in Table 2 found that two articles were high quality, 11 were good quality, six were moderate quality and one was low quality.

**TABLE 1:** Included study characteristics and summary.

Study (lead author and date)	Country	Setting	Timeframe	Population	Design	Total patients^a^ (*n*): total interventions^b^ (*n*)	Type of interventions (*n* if available)	Major findings
Anshu 2023 [12]	India	Single peripheral military hospital	August 2020 to December 2022	Aged 5–88 years. CT confirmed TBI and findings of secondary intracerebral injury or clinical signs of deterioration	Retrospective case series	23:23	17 decompressive craniectomies 4 burr hole evacuations 2 EVDs	All patients had a pre‐operative CT General surgeon operated Injuries: ○ 56.52% SDH ○ 30.43% EDH ○ 17.39% haemorrhagic contusions ○ 8.69% intraventricular bleed with obstructive hydrocephalus 26% (*n* = 6) hospital mortality rate Long‐term follow‐up: ○ 4% (*n* = 1) were GOSE1 ○ Average GOSE score of 30.43% ○ Nil post‐operative complications or deficits in other patients
Attebury 2006 [13]	Tanzania	Single centre	January 2006 to September 2007	Patients receiving neurosurgical intervention for any indication at the hospital by either a neurosurgeon or general surgeon	Retrospective case series	18:NS	Overall rates^c^: 7 burr holes 11 craniotomies 4 skull fracture repairs	Did not specify if patients had a pre‐operative CT General surgeon operated Post‐operative status at follow‐up of all neurosurgical procedures (including non‐TBI) ○ 14 patients were deceased ○ 15 were living ○ 14 were lost to follow‐up and 3 had their record unavailable. These procedures were compared to those performed by a United States neurosurgeon with comparable outcomes
Bishop 2006 [14]	Australia	Multiple centres	1997 to 2001	General surgeons (*n* = 161)	Survey	NS:~600	37% were burr holes 41% were craniotomies.	Did not specify if patients had a pre‐operative CT General surgeon operated The frequency of procedures increased with distance from a neurosurgical centre (*p* < 0.0001)
Deskit 2022 [15]	India	Multicentre	November 2017 to November 2020	Patients with a TBI, aged 33–77 years old.	Case series	7:8	2 burr holes 5 craniectomies	All patients had pre‐operative CT General surgeon operated Injuries: ○ 57% EDH ○ 43% SDH 14% mortality 86% patients survived with minimal disability 1 patient required re‐operation for a rebleed
Fischerstrom 2014 [16]	Sweden	Multicentre	2005–2010	Patients referred to the neuro‐intensive care unit in Uppsala after acute evacuation of intracranial haematomas in the regional hospitals	Retrospective case series	49:75	Not defined	All patients had pre‐operative CT General surgeon operated Injuries: ○ 35% EDH ○ 65% SDH Mortality 18% at follow‐up (6–26 months post intervention) 31% (*n* = 15) required reoperation within 24 h, and a further 22% (*n* = 11) did within 3 weeks. Postoperative CT scan was improved in 92% of the patients and unchanged in 8% Long‐term outcomes: ○ 51% GOSE ≥ 5 ○ 33% GOSE ≤ 4 ○ 16% GOSE unknown
Gilligan 2017 [17]	Australia	Single centre	January 2000 to January 2013	Patients admitted to a neurosurgical hospital from a rural centre	Retrospective case series	9:9	Burr holes and craniectomies	All patients had pre‐operative CT General surgeon operated Injuries: ○ 44.4% EDH ○ 44.4% SDH ○ 11.1% combined EDH and SDH 22% of cases had neurosurgeons assisting in the procedure 11% mortality Long‐term outcomes in survivors: ○ 50% GOS 5 ○ 50% GOS 4
Havill 1998 [18]	New Zealand	Single centre	July 1987 to July 1997	Patients admitted to ICU.	Retrospective case series	151:151	Burr holes and craniectomy	All patients had pre‐operative CT General surgeon operated 31% (*n* = 47) were transferred to a neurosurgical centre 29% of those transferred died at the neurosurgical centre
Howard 2020 [19]	Ireland	Single centre	Not specfied	2 patients with CT confirmed TBI, aged 32 and 31 years old	Retrospective case series	2:2	2 burr holes	All patients had pre‐operative CT ED consultant operated Both were transferred via ambulance to a neurosurgical unit for a craniectomy, and required further decompression of burr hole with suction on route Patient 1 had a GOS 5, whilst patient 2 had normal cognitive ability (nil GOS reported)
Hu 2022 [20]	Cambodia	Single centre	January 2015 to December 2016	TBI receiving emergency surgical intervention	Prospective case series.	235:235	28 burr holes 207 craniotomies	All patients had pre‐operative CT General surgeon operated Mortality 7.2% overall ○ 7% EDH ○ 10.8% SDH 92.8% (*n* = 218) patients experienced favourable outcomes (GOS > 3) at 3 months post‐intervention. Preoperative GCS < 7 was associated with an unfavourable outcome at 3 months after injury (OR 26.3, 95% CI 7.9–87.1)
Kelly 2024 [21]	Australia	Multicentre	January 2001 to December 2022	Patients who underwent an emergency surgical intervention at Queensland hospitals without an onsite neurosurgical service	Retrospective cohort study	22:23	4 burr holes 19 craniectomies or craniotomies	All patients except 2 had a pre‐operative CT. If they survived the procedure, they had a follow‐up CT. General surgeon operated Injuries: ○ 41% EDH ○ 59% SDH Mortality 55% overall ○ 22% for EDH ○ 77% for SDH GOS 5 was 33% after an EDH and 8% after a SDH. Patients who received burr hole only had no evidence of radiological improvement ○ 50% mortality in subgroup ○ Survivors required re‐operation
Leitgeb 2012 [22]	Austria, Croatia and Slovakia	Multicentre	January 2001 to December 2005	Patients admitted to ICUs with a GCS of ≤ 8	Prospective case series	120:148	61 craniotomies 31 craniectomies 23 decompressions	All patients had pre‐operative CT Trauma surgeon operated 23% (*n* = 28) required revision surgery Hospital mortality was 40.8%, compared to 39.3% in those treated by a neurosurgeon (*p* = 0.81) At 12 months, GOS ≥ 4 was 43.3% in the trauma surgeon cohort and 35.6% in the neurosurgeon cohort (*p* = 0.19)
Luck 2015 [23]	Australia	Single centre	January 1, 2008 to December 31, 2013	All emergency neurosurgery patients	Prospective case series	161:195	44 burr holes 49 craniectomies 37 craniotomies 32 EVDs 9 posterior fossa decompressions	All patients had pre‐operative CT General surgeon operated Injuries ○ 26.7% SDH ○ 16.4% acute on chronic SDH ○ 5.6% EDH ○ 3.1% depressed skull fracture ○ 2.5% chronic SDH 14% (*n* = 28) patients required surgical re‐intervention 23% 30‐day mortality in head trauma patients. The head injury severity correlated to the Glasgow Outcome Scale (*R* ^2^ = 0.12, *p* < 0.001). Other factors associated with worse surgical; included remote location of injury (*p* = 0.022), time from injury to operation more than 24 h (*p* = 0.023) and the specific neurosurgical diagnoses (*p* = 0.004).
Raman 2023 [24]	Australia	Multicentre	n/a	Surgical theatre nurses or service directors from rural and regional Queensland hospitals with a CT scanner and are not within 2 h of a tertiary centre	Survey	n/a	n/a	26 responses from eligible hospitals, in which 69.2% of hospitals (*n* = 18) had surgical services. 42% (*n* = 11) of respondents had complete emergency cranial access kits, 19% (*n* = 5) had incomplete kits. 38% (*n* = 10) had no kit or were unsure about whether they had one 7.7% of responding hospitals reported using the equipment in the last 12 months, with 19.2% using it in the last 10 years
Rinker 1998 [25]	United States of America	Single centre	January 1, 1991, to April 1 1997	Patients with TBI deemed too unstable for transport before decompression	Prospective case series	8:8	8 craniectomies	All patients had pre‐operative CT General surgeon operated Injuries: ○ 62.5% EDH ○ 25% SDH ○ 12.5% combined EDH and SDH Mortality 12.5% (*n* = 1, patient with SDH). All discharged patients had a GOS ≥ 4 at the mean follow‐up of 3.6 years.
Simpson 1984 [26]	Australia	Single site	August 29, 1981 to February 26, 1982	Consecutive patients with head or spinal injuries transferred to the major hospital from rural and regional centres	Prospective case series	3:3	3 craniotomies	No CT was available Local medical officer operated In‐patient mortality 67% (*n* = 2) Surviving patient had “considerable disability”
Treacy 2005 [27]	Australia	Single centre	January 1992 and June 2004	Patients who underwent an emergency neurosurgical procedure	Prospective case series	124:147	9 burr holes 115 craniotomies	Injuries: ○ (*n* = 27) EDH ○ 81 acute SDH ○ 16 ICH Nil imaging specified General surgeon operated 18.5% (*n* = 23) required repeat surgery Mortality at 3 months: ○ 9% EDH ○ 2% chronic SDH ○ 44% acute SDH ○ 77% for ICH GOS ≥ 4 at 3 months: ○ 82% EDH ○ 84% chronic SDH ○ 45% acute SDH ○ 14% ICH
Umo 2023 [28]	Papua New Guinea	Multicentre	1 December 2018 and 30 April 2022	Patients with moderate to severe TBI	Retrospective case series	39:39	32 burr hole and craniotomies 7 craniectomies	Nil imaging specified General surgeon or local medical officer operated Mortality^d^: ○ 14.3% bur hole and craniotomy ○ 16.6% craniectomy
Visvanathan 1994 [29]	Malaysia	Single‐centre	n/a	Severe head injuries during the 29‐month study period	Retrospective case series	40:46	Craniotomy or craniectomy.	All patients had an x‐ray, and some received a CT General surgeon operated Injuries ○ 50% EDH ○ 20 SDH ○ 15% intracranial haemorrhage 49% overall mortality at follow‐up (mean period 7.06 months) 15% (*n* = 6) reoperation. Mortality by subtype: ○ 15% EDH ○ 87.5% SDH ○ 50% ICH After follow‐up (mean 7 months from discharge), GOS 5 in: ○ 55% of EDH ○ 12.5% of SDH ○ 0% of ICH 25% of patients had surgical reintervention for recurrent bleeding or residual clot detected by CT scans
Winkler 2010 [30]	Tanzania	Single centre	2003	Patients with neurologic or neurosurgical disorders	Prospective case series	7:7	3 burr holes 4 depressed fractures elevated	Some patients had x‐rays General surgeon operated All burr holes were indicated for SDH Mortality was 0% Neurological sequelae (non‐specified) were reported in all patients who had burr holes
Yusof 2021 [31]	Australia	Multicentre	n/a	Nurse unit manager or general surgical registrars of regional and rural hospitals that provide surgical services in New South Wales.	Survey	n/a	n/a	41% (*n* = 23) owned a Hudson brace, perforator and burr hole. 70% of hospitals with equipment, store it sterile in an operating theatre. 20% of responding hospitals had used the equipment within the last 10 years. 45% reported mortality at the time of discharge (although status of 36% was unavailable). Of those that arrived to a neurosurgical centre, mortality was 33%. No further information available on patient outcomes.

*Note:* All included studies were examined for their setting, timeframe, population, design, number of patients operated on and the total interventions by a non‐neurosurgeon, type of interventions, whether patients received CT scans, the professional who performed the surgery, and patient outcomes defined as mortality and neurological measures at the latest defined period.

Abbreviation: NS, not specified.

^a^ Receiving operative intervention by a non‐neurosurgeon.

^b^ By a non‐neurosurgeon.

^c^ Did not specify whether procedures were performed by the general surgeon or the neurosurgeon.

^d^ Data obtained from an unpublished correction.

**TABLE 2:** Quality assessment of included articles via QATSDD.

Article criteria	1	2	3	4	5	6	7	8	9	10	11	12	13	14	15	16	Total score	%^a^
Anshu [12]	1	3	2	0	2	3	1	2	1	2	—	2	1	—	0	2	22	52.4
Attebury [13]	3	1	3	1	2	3	1	3	0	3	—	3	1	—	0	2	24	57
Bishop [14]	3	3	3	0	3	3	2	3	1	3	—	3	3	—	0	3	30	71
Deskit [15]	2	2	3	0	1	2	1	2	0	2	—	2	0	—	0	1	18	42.9
Fischerstrom [16]	3	3	3	0	3	3	1	3	0	3	—	3	1	—	1	3	30	71.4
Gilligan [17]	2	2	2	0	3	2	1	2	0	1	—	1	0	—	0	0	16	38.1
Havill [18]	2	2	3	0	2	3	0	3	0	3	—	2	0	—	0	0	20	47.6
Howard [19]	2	1	2	0	1	0	0	0	0	1	—	1	0	—	0	0	8	19
Hu [20]	3	3	3	1	2	3	1	2	3	3	—	3	1	—	1	3	32	76.2
Kelly [21]	3	3	1	1	3	1	2	3	2	3	—	3	3	—	1	2	31	73.8
Leitgeb [22]	3	3	3	0	2	3	2	3	2	2	—	2	3	—	0	2	30	71.4
Luck [23]	2	2	2	0	3	3	3	3	3	2	—	2	3	—	1	2	31	73.8
Raman [24]	2	2	3	1	2	3	2	3	1	2	—	2	2	—	2	2	29	69
Rinker [25]	3	1	2	1	2	2	1	3	0	2	—	2	0	—	1	1	21	50
Simpson [26]	2	2	2	0	1	1	1	2	1	2	—	2	1	—	0	0	17	40.5
Treacy [27]	2	3	3	0	3	3	3	3	2	3	—	3	3	—	0	1	32	76.2
Umo [28]	3	3	3	0	1	3	1	3	2	2	—	3	1	—	0	2	27	64.3
Visvanathan [29]	1	0	1	0	1	2	1	1	0	1	—	2	1	—	0	0	11	26.2
Winkler [30]	3	1	3	0	2	0	0	2	1	2	—	2	1	—	1	0	18	42.9
Yusof [31]	3	3	3	1	2	3	1	2	0	2	—	2	1	—	0	1	24	57.1

*Note:* The quality assessment was independently conducted and agreed upon by L.B. and E.J. The corresponding numerical criteria and scoring system of QATSDD is explained in Appendix S3.

^a^ Article's score divided by total possible score (42) × 100.

### 3.2 Setting

The studies were set across 13 countries, with only one completed in multiple countries [22]. Eight studies were conducted in Australia [14, 17, 21, 23, 24, 25, 27, 30], two each in India [12, 15], and Tanzania [13, 30], with single projects in Ireland [19], Cambodia [20], United States of America [25], Papua New Guinea [28], Sweden [16], New Zealand [18], and Malaysia [29]. All were set in non‐metropolitan hospitals remote from neurosurgical centres, hence justifying intervention by non‐neurosurgeons.

Three studies defined the distance in kilometres to definitive neurosurgical care from the non‐metropolitan treating hospital [15, 18, 27]. Distances to the closest neurosurgical centre significantly ranged, being 130 km from Waitkato [18], 435 km from Leh to Srinagar [15], to 2600 km from Darwin (which had no neurosurgical centre within the state), to Adelaide [27]. Following surgical intervention, seven studies transferred patients to a neurosurgical centre [17, 18, 19, 21, 25, 26], six studies managed patients onsite until discharge [13, 14, 15, 27, 28, 29, 30], three studies only transferred complex cases [14, 22, 23], and two studies did not define the location of post‐operative management [12, 20]. Of the studies that continued management onsite, two cited state‐wide resource limitations [13, 27], and one reported geographical barriers as reasons for not transferring [15].

### 3.3 Interventions

The types of procedures explored included burr holes, craniectomies, craniotomies, extraventricular drains (EVDs) and skull fracture elevations. Indications included EDHs, SDHs, intraventricular bleeds with/without obstructive hydrocephalus and skull fractures. Of the 18 included interventional studies, burr holes were performed in 13, craniectomies in 12, craniotomies in 10 and ventricular drains in three. In the largest sample size, which approximated 600, the most common procedure was craniotomies (41%) [14]. Pre‐operative CT scans were performed in 65% of interventional studies. In the five that did not report on the use of CT scans prior to surgery, three were in Australia [14, 26, 27], two were in Tanzania [13, 30], and one in Papua New Guinea [28]. Two of these sites reported that they did not have access to CT [26, 30], whilst the remaining three did not specify [13, 14, 27].

### 3.4 Clinicians and Their Support

In the 18 interventional studies, the majority (14) had procedures performed by general surgeons, with a mixture of an Emergency Specialist, Trauma Surgeon and unspecified clinicians in the remaining four articles [19, 22, 26, 30]. Remote clinicians accessed neurosurgical advice to varying extents in 11 studies [12, 13, 14, 15, 16, 17, 18, 22, 25, 26, 27]. Neurosurgeon involvement ranged from providing approval prior to all interventions [16, 25], delivering advice via telehealth [22], which in one study was utilised only in complex cases [18], and observing procedures with live transmitted guidance as required [14, 25]. One study assessed rural doctors' access to telecommunication with neurosurgeons, with 61%–81% of doctors stating that they ‘never’ or ‘rarely’ experienced delays in receiving urgent neurosurgical tele‐advice [14].

### 3.5 Patient Outcomes

Patient outcomes were reported heterogeneously, with measures including mortality, improvement on CT and functional outcomes. In‐hospital mortality and follow‐up mortality were often documented without measured time periods. Mortality rates ranged from 0% (two small studies with two and seven patients respectively) [19, 30], through to 67% in a study with three patients [26]. Most studies did not define patients' causes of death. Of those that did, one study reported that TBI was the cause of death in all nine patients [16]. Others reported mortality aetiologies related to surgery, including intraoperative cardiac arrest [15], anaesthesia, possible sepsis and those unrelated, including pneumonia [14]. One study performed statistical sub‐analyses of the mortality rate data against several variables, finding reduced mortality in patients with reactive pupils (OR: 0.02, 95% CI: 0.00–0.17, *p* = 0.0005) and a higher GCS (OR: 0.77, 95% CI: 0.63–0.95, *p* = 0.0147) [28]. Five articles comparing mortality against TBI type found increased mortality in SDHs compared to EDHs [20, 21, 23, 25, 27]. Notably, Treacy et al. [27] reported a 3‐month mortality of 44% for acute SDHs compared to 9% in EDHs. Two studies compared patient mortality following neurosurgeon to non‐neurosurgeon intervention and found no statistical difference [13, 22]. Two studies measured operative success with repeated CT scans, with radiological improvement ranging from 55% to 82% in most interventions, except for burr holes, which showed no changes [16, 21].

Post‐surgical functional outcomes were described in 14 articles. The Glasgow outcome scale (GOS) was used in eight studies [17, 20, 21, 22, 23, 24, 25, 27, 29], with follow‐up periods ranging from discharge [21], a median of 3.6 years [25], and unspecified in two [17, 22]. The Glasgow Outcome Scale Extended (GOSE) was used in two articles [12, 16]. Of the other studies, non‐specific comments such ‘cognitively normal’ [19], ‘neurological sequelae’ [30], and ‘no motor sensory deficit’, ‘neuropsychiatric complication’ and ‘minimal motor deficit’ [15] were used.

### 3.6 Complications

Post‐operative complications were variably recorded over inconsistent intervals and with limited details. Ten of the interventional articles described post‐operative complications, with rebleeds requiring reoperation being the most common [12, 13, 15, 16, 21, 22, 23, 27, 28, 29]. Multiple studies noted that patients required re‐operation due to patient deterioration, by re‐opening the site to perform further decompression by using irrigation and suction. This was performed either by the general surgeon prior to tranfer [15, 27], during inter‐hospital transfer within an ambulance [19], upon arrival with the neurosurgeon [21], or did not specify the setting [16, 22, 29]. Rates of neurosurgeon re‐operation ranged from 0% in those who survived [12], to 100% in a small study of two patients [19]. One study comparing a trauma surgeon to a neurosurgeon found higher rates of re‐operation in trauma surgeons (23.3% compared to 12.0%, *p* = 0.012). It is unclear if patients requiring reoperation have a higher mortality (41.2% vs. 39.9%; *p* = 0.88) [22]. Post‐operative complications increased patients' risk of death (OR: 5.25, *p* = 0.0133) [28].

### 3.7 Preparedness

The three included surveys examined facilities' preparation for neurosurgical intervention by non‐neurosurgeons, in terms of hospitals having the appropriate equipment for procedures and doctors' self‐reported confidence. In the 56 non‐metropolitan New South Wales' hospitals surveyed, 41% had the necessary surgical equipment [31]. In a similar study, 42% of responding non‐neurosurgical hospitals in Queensland were equipped to perform an emergency craniectomy [24]. In the past decade, 20% and 19.2% of the respective hospitals had used the equipment [24, 31]. Rural Surgeons' confidence to perform a burr hole increased with distance from a neurosurgical centre (*p* = 0.015) [14].

## 4 Discussion

This scoping review examined the practise of non‐neurosurgeons performing emergent neurosurgical intervention for acute TBIs in nonmetropolitan environments. From 20 studies, 17 of which were interventional and included over 2000 surgical interventions in 13 countries, it was most commonly general surgeons performing burr holes, craniectomies and craniotomies on patients with CT‐confirmed lesions. The surveys met inclusion criteria and provided valuable insight about the procedure's retrospective frequency and logistics of its implementation, including equipment availability.

Resourcing challenges were a theme in the included studies. Nearly half of the studies were in low‐income countries, [32] where the largest barriers included costs of care, lack of equipment, inadequate health infrastructure and limited access to neurosurgeons [33]. The extent of this is exemplified in one Indian study, where the surgeon used personal funds to purchase haemostatic agents for surgery to overcome this barrier [15]. In high‐income countries like Australia, large distances and retrieval times challenged the provision of timely neurosurgical care. A Western Australian study reported a median transfer time for major rural trauma cases transported to the major trauma hospital of 9.2 h [34], which significantly exceeds national recommendations for TBIs to reach a neurosurgeon within 2 h of injury [8]. Despite healthcare in Australia being well funded, resource availability was another logistical challenge to non‐neurosurgical centres providing surgical intervention for TBIs, with less than half of the responding hospitals in both Australian surveys having appropriate emergency neurosurgical equipment [24, 31]. Despite this, non‐neurosurgeons' confidence to perform a decompression increased with distance from a neurosurgical centre [14], likely reflecting the resilience of remote centres to the tyranny of distance.

General and trauma surgeons performed most of the surgical interventions. Two studies did not define the qualifications of the medical doctors [26, 28], and one confirmed an Emergency Physician [19]. Qualified surgeons performing time‐critical decompression in non‐metropolitan centres is not a surprising result, but the few studies where other clinicians were required to operate is notable. In Australia, most non‐metropolitan facilities' senior staffing consists of a combination of Emergency Physicians, Rural Generalists and General Practitioners, as well as specialty registrars and International Medical Graduates. Currently, only the Australian College of Rural and Remote Medicine (ACRRM) requires its Fellows to perform burr holes [35, 36, 37, 38]. However, the Australasian College for Emergency Medicine (ACEM) and Prehospital and Retrieval Medicine (PHRM) requires its graduates to complete resuscitative thoracotomies [35, 36], which is arguably more complex than a burr hole. Of note, ACRRM and the Royal Australian College of General Practitioners (RACGP) do not require their fellows to have that skill [35, 38]. Whilst case reports were omitted from this review, various articles describe the potential feasibility of non‐surgeons performing emergent decompression for severe TBIs. Two case reports at different hospitals without onsite neurosurgical services described Emergency Physicians utilising an intraosseous needle for trephination to facilitate their patient's recovery without neurological deficit [39, 40]. Similarly, a General Practitioner on a remote island in Japan successfully performed a burr hole using a makeshift device [41]. With appropriate training, equipment and governance, there may be a role for General Practitioners, Rural Generalists, Emergency Physicians and PHRM specialists performing decompression of severe TBIs in emergent situations. With distance from neurosurgical facilities identified as a driver for non‐neurosurgeons performing interventions, in Australasia, these specialists are likely to be with the patient earlier, and when timely intervention may improve outcomes, training, equipping and supporting those specialists may save lives.

A recurrent theme within the literature was remote neurosurgical support provided to the non‐neurosurgeons. Telehealth infrastructure facilitated CT transmission, live audio and sometimes video calls [12, 13, 14, 15, 16, 17, 18, 22, 25, 26, 27]. Telehealth use in Australasian healthcare has rapidly expanded over recent years [42]. With its increased presence and use, telemedicine for neurosurgical consultation in emergencies is a life‐saving, time‐efficient and cost‐effective recommendation from the World Society of Emergency Surgery [43]. The majority of the Australian rural surgeons surveyed reported that they were able to access remote neurosurgical support in emergency settings [24, 31]. Given the benefits of neurosurgeons supporting doctors in remote locations, it is also important to establish and maintain a high‐functioning telehealth system to deliver the best outcomes for patients with severe TBIs.

Over 2000 procedures were described across the 18 unique interventional studies. The most common interventions were burr holes, followed by craniectomies and craniotomies. A level IIA recommendation in severe TBI management is for a large frontotemporal decompressive craniectomy to reduce mortality and improve neurologic outcomes [44]. Burr holes can be considered a simplified alternative for a decompressive craniectomy, particularly in under‐resourced hospitals [45], reflected by their higher prevalence in this review. In the one study that compared outcomes between burr holes and craniectomies, burr holes were considered less efficacious [21]. Whilst burr holes were the most common procedure done, likely due to their simplicity compared to other procedures, their efficacy compared to other approaches is unclear. Notably, no studies compared a burr hole by a non‐neurosurgeon against transfer and delayed access to a neurosurgeon. Whilst formal decompression by a neurosurgeon remains the gold standard, a burr hole performed several hours earlier by a local clinician can relieve raised ICP in severe TBIs to optimise patient outcomes.

All neurosurgical interventions were performed for EDHs, SDHs, intraventricular bleeds and skull fractures. In most studies, a CT scan was performed prior to surgery. Three studies did not specify whether it was used [13, 14, 27]; however, it is likely that patients were imaged to determine the TBI type and guide the need for emergent intervention. Two studies did not have access to a CT scanner so relied on clinical signs of deterioration to indicate the need for intervention [26, 30]. A CT scan is recommended prior to neurosurgical intervention because without it, there is increased risk of inaccurate localisation of the pathology [46]. Although an Australian study reported that eight out of the 11 responding remote hospitals had access to 24‐h CT, intensive care unit and ability to care for ventilated patients, this survey was specifically sent to surgeons, and facilities staffed with surgeons are likely to have these resources [14]. In our analysis, given the high proportion of the included articles that presented interventions performed by surgeons, it is therefore unsurprising that most had a CT performed prior. Those in non‐metropolitan Australia have significantly reduced access to radiological services, with those in rural and remote Australian towns often lacking access to CT [47]. Waiting for a CT scan may delay critical interventions with worse outcomes; however, this needs to be balanced against performing an invasive procedure without confirmed lesions. The Brain Trauma Foundation guidelines for ‘the Management of Acute Neurotrauma in Rural and Remote Locations’ recommends commencing burr hole exploration of suspected traumatic intracranial haemorrhages by local medical officers if a patient is deteriorating and transfer to a neurosurgeon is unavailable within 2 h [8]. This practise was implemented by Simpson et al. [26] with mortality in two out of three patients. Contrastingly, all three patients who had burr holes without prior imaging for SDH survived in the report by Winkler et al. [30]. Limited literature exists about neurosurgical procedures on TBI patients with clinically raised ICP without prior CT. Research is needed to explore the need and feasibility versus potential risks of surgical interventions for TBI in regions without a CT.

Patient‐centred outcomes were inconsistently reported. Mortality and multiple measures of morbidity were presented, but often incompletely variably. Mortality rates varied between different studies, but did not exceed 67% [26]. Patients with SDHs had a greater mortality than EDHs [20, 21, 23, 25, 27], consistent with existing literature from neurosurgeon‐performed interventions of these injuries [48]. The two studies that compared neurosurgeons to non‐specialist surgeons found no statistical difference in mortality [13, 22]. There was minimal sub‐analyses of mortality influences, but variables reported included GCS on presentation, haemorrhage severity on CT, and patient comorbidities [12, 15, 16, 17, 18, 20, 23, 28]. Significant variables included pre‐injury warfarin use [16], remote geographical location, and time from injury to operation exceeding 24 h [23]. The latter two support efforts to identify and instigate system improvements that could lead to expedited decompression of severe TBI. Functional outcomes were reported over variable timeframes, potentially underestimating the benefits of non‐neurosurgeons performing emergent decompression since TBI patients' functional outcomes can improve 12 months following their injury [49]. The inconsistent reporting across the included literature makes interpretation of the mortality and morbidity benefits challenging, and future research should include consistent and established measures. Similarly, the heterogeneity in the study designs and small sample sizes makes it unfeasible to determine whether patient outcomes have improved over time with potential advancements in care.

Our study has several limitations. Many studies had small sample sizes, reducing the statistical significance and reliability of findings [50]. Including retrospective designs limited the completeness and accuracy of the data. Despite this, 65% of articles were considered ‘good’ or ‘reasonable’ quality using the QATSDD measure. Larger sample sizes and prospective designs in future research would enhance the quality of evidence. The research included both high‐income and low‐income countries, with the differing resources and staff training likely influencing patient outcomes. The literature also spans 39 years, with more recent studies likely to have greater access to neurosurgical resources, telecommunication services and faster patient transfer networks. However, despite medical advances, a recent literature review reported that patient outcomes have not significantly improved following craniectomy for a TBI historically [51]; thus, the timeframe of included studies is not expected to bias our results. Whilst remote location is an important factor in this study, very few projects clearly defined the non‐metropolitan hospitals' location, supporting facilities and patient transfer services sufficiently for this to be described and discussed. Despite reasonable efforts including reference checks to find all applicable articles and reviewing additional terms in articles identified, it is possible that relevant articles have been missed. Leading causes of health‐related literature not being published include negative findings or statistically insignificant results [52]. This potential publication bias can lead to poor replicability of results and insufficient conclusions in literature reviews [53].

## 5 Conclusion

Emergency neurosurgical intervention by a non‐specialist doctor for patients with severe TBIs may be lifesaving for patients without timely access to a neurosurgical centre. The existing literature focuses on general surgeons performing burr holes on patients with a CT‐confirmed EDH and SDH in settings remote from neurosurgical care, but the mortality and morbidity benefits are unclear. This practise appears to be feasible; however, further efforts are required to develop the capacity of non‐neurosurgical facilities to perform these procedures, by strengthening telehealth networks and providing appropriate equipment resourcing. The approach of non‐neurosurgeons performing surgical interventions on severe TBIs could also apply to specialists on aeromedical retrievals, where long delays are commonplace. Further research is urgently required to examine typical timeframes for retrieving TBI patients from non‐metropolitan areas. If timely neurosurgical care is unobtainable, our current study suggests that non‐neurosurgeons performing surgical interventions for these patients may be the solution to providing the lifesaving, time‐critical care.

## Author Contributions

Conception and design initiated by C.G. Research question, methods, data collection, results interpretation and manuscript writing completed by L.B. and C.G. Data analysis by L.B., E.J. and C.G. Article drafted and revised critically for intellectual content and final approval of the version to be published by L.B., C.G. and G.D.

## Conflicts of Interest

The authors declare no conflicts of interest.

## Supporting information


**Appendix S1.** PRSIMA extension for scoping reviews checklist.
**Appendix S2.** Search strategy.
**Appendix S3.** QATSDD criteria and scoring [10].

## Data Availability

Data sharing is not applicable to this article as no new data were created or analyzed in this study.
