# Supplementary material for: Can Non‐Neurosurgeons Operate on Traumatic Brain Injuries in Non‐Metropolitan Areas? A Scoping Review
Source: Emerg Med Australas. 2025 May 21;37(3):e70055. doi: 10.1111/1742-6723.70055 (PMC12095900; doi:10.1111/1742-6723.70055)
Supplement: Supplementary file 1 — Appendix S1. PRSIMA extension for scoping reviews checklist. Appendix S2. Search strategy. Appendix S3. QATSDD criteria and scoring [10]. [file EMM-37-0-s001.docx]

**Appendices**

**Appendix 1 – PRSIMA extension for Scoping Reviews Checklist**

| **SECTION** | **ITEM** | **PRISMA-ScR CHECKLIST ITEM** | **REPORTED ON PAGE #** |
| --- | --- | --- | --- |
| **TITLE** | | | |
| Title | 1 | Identify the report as a scoping review. | Cover, 1 |
| **ABSTRACT** | | | |
| Structured summary | 2 | Provide a structured summary that includes (as applicable): background, objectives, eligibility criteria, sources of evidence, charting methods, results, and conclusions that relate to the review questions and objectives. | 1 |
| **INTRODUCTION** | | | |
| Rationale | 3 | Describe the rationale for the review in the context of what is already known. Explain why the review questions/objectives lend themselves to a scoping review approach. | 2-3 |
| Objectives | 4 | Provide an explicit statement of the questions and objectives being addressed with reference to their key elements (e.g., population or participants, concepts, and context) or other relevant key elements used to conceptualize the review questions and/or objectives. | 3 |
| **METHODS** | | | |
| Protocol and registration | 5 | Indicate whether a review protocol exists; state if and where it can be accessed (e.g., a Web address); and if available, provide registration information, including the registration number. | 3 |
| Eligibility criteria | 6 | Specify characteristics of the sources of evidence used as eligibility criteria (e.g., years considered, language, and publication status), and provide a rationale. | 3-4, Figure 1 |
| Information sources* | 7 | Describe all information sources in the search (e.g., databases with dates of coverage and contact with authors to identify additional sources), as well as the date the most recent search was executed. | 3, Figure 1 |
| Search | 8 | Present the full electronic search strategy for at least 1 database, including any limits used, such that it could be repeated. | Appendix 2 |
| Selection of sources of evidence† | 9 | State the process for selecting sources of evidence (i.e., screening and eligibility) included in the scoping review. | 3-4, Figure 1 |
| Data charting process‡ | 10 | Describe the methods of charting data from the included sources of evidence (e.g., calibrated forms or forms that have been tested by the team before their use, and whether data charting was done independently or in duplicate) and any processes for obtaining and confirming data from investigators. | 3-4 |
| Data items | 11 | List and define all variables for which data were sought and any assumptions and simplifications made. | 4 |
| Critical appraisal of individual sources of evidence§ | 12 | If done, provide a rationale for conducting a critical appraisal of included sources of evidence; describe the methods used and how this information was used in any data synthesis (if appropriate). | 4 |
| Synthesis of results | 13 | Describe the methods of handling and summarizing the data that were charted. | 3-4 |
| **RESULTS** | | | |
| Selection of sources of evidence | 14 | Give numbers of sources of evidence screened, assessed for eligibility, and included in the review, with reasons for exclusions at each stage, ideally using a flow diagram. | 3-5 Figure 1 |
| Characteristics of sources of evidence | 15 | For each source of evidence, present characteristics for which data were charted and provide the citations. | 4-9,Table 1 |
| Critical appraisal within sources of evidence | 16 | If done, present data on critical appraisal of included sources of evidence (see item 12). | 4, Table 2 |
| Results of individual sources of evidence | 17 | For each included source of evidence, present the relevant data that were charted that relate to the review questions and objectives. | Table 1 |
| Synthesis of results | 18 | Summarize and/or present the charting results as they relate to the review questions and objectives. | 5-9,Table 1 |
| **DISCUSSION** | | | |
| Summary of evidence | 19 | Summarize the main results (including an overview of concepts, themes, and types of evidence available), link to the review questions and objectives, and consider the relevance to key groups. | 7-10, Table 1 |
| Limitations | 20 | Discuss the limitations of the scoping review process. | 9-12 |
| Conclusions | 21 | Provide a general interpretation of the results with respect to the review questions and objectives, as well as potential implications and/or next steps. | 13-14 |
| **FUNDING** | | | |
| Funding | 22 | Describe sources of funding for the included sources of evidence, as well as sources of funding for the scoping review. Describe the role of the funders of the scoping review. | 15 |

JBI = Joanna Briggs Institute; PRISMA-ScR = Preferred Reporting Items for Systematic reviews and Meta-Analyses extension for Scoping Reviews.

* Where *sources of evidence* (see second footnote) are compiled from, such as bibliographic databases, social media platforms, and Web sites.

† A more inclusive/heterogeneous term used to account for the different types of evidence or data sources (e.g., quantitative and/or qualitative research, expert opinion, and policy documents) that may be eligible in a scoping review as opposed to only studies. This is not to be confused with *information sources* (see first footnote).

‡ The frameworks by Arksey and O’Malley (6) and Levac and colleagues (7) and the JBI guidance (4, 5) refer to the process of data extraction in a scoping review as data charting*.*

§ The process of systematically examining research evidence to assess its validity, results, and relevance before using it to inform a decision. This term is used for items 12 and 19 instead of "risk of bias" (which is more applicable to systematic reviews of interventions) to include and acknowledge the various sources of evidence that may be used in a scoping review (e.g., quantitative and/or qualitative research, expert opinion, and policy document).

**Appendix 2 – Search Strategy**

| **Database searched** | **Search strategy** |
| --- | --- |
| CINAHL | 1. (MH “head injuries+”) OR (MH “trauma+”) OR (MH “trauma nurses”) OR (MH “trauma centres”) OR (MH “registries, trauma”) OR (MH “trauma nursing”) 2. (MM “craniotomy”+ ”) OR (MH “decompressive craniectomy”) 3. (MH “hospitals, rural) OR (MH “rural population) OR (MH “rural nursing) OR (MH “rural areas”) OR (MH “rural health”) OR (MH “rural health personnel) OR (MH “rural health centres”) OR (MH “rural health services”) OR (MH “Australian rural nurses and midwives”) OR (MH “association for Australian rural nurses”) OR (MH “Association for Australian rural nurses”) OR (MH “services for Australian rural and remote allied health”) 4. "craniocerebral injuries" OR "craniocerebral injury" OR "craniocerebral trauma" OR "craniocerebral traumas" OR "crushing skull injuries" OR "crushing skull injury" OR "forehead trauma*" OR "frontal region trauma*" OR "head injur*" OR "head trauma*" OR "occipital region trauma*" OR "occipital trauma*" OR "parietal region trauma*" OR “brain trauma*” OR “brain injur*” OR “brain laceration*” OR “epidural hemorrhage*” OR “epidural hematoma*” OR “subdural hemorrhage*” OR “subdural hematoma*” OR “intracranial hemorrhage*” OR “intracranial hematoma*” OR “brain injur*” OR “epidural haemorrhage*” or “epidural haematoma*” OR “subdural haemorrhage*” OR “subdural haematoma*” OR “intracranial haemorrhage*” OR “intracranial haematoma*” OR “intracranial hematoma*” 5. “craniectom*” OR “craniotom*” OR "burr hole*" OR “trephin*” OR “trepan*” OR “Brain surg*” 6. rural* OR remote* 7. 1 OR 4 8. 2 OR 5 9. 3 OR 6 10. 7 AND 8 AND 9 |
| CINAHL (additional search) | 1. (MH “head injuries+”) OR (MH “trauma+”) OR (MH “trauma nurses”) OR (MH “trauma centres”) OR (MH “registreies, trauma”) OR (MH “trauma nursing”) 2. (MM “craniotomy”+ ”) OR (MH “decompressive craniectomy”) 3. (MH “hospitals, rural) OR (MH “rural population) OR (MH “rural nursing) OR (MH “rural areas”) OR (MH “rural health”) OR (MH “rural health personnel) OR (MH “rural health centres”) OR (MH “rural health services”) OR (MH “Australian rural nurses and midwives”) OR (MH “association for Australian rural nurses”) OR (MH “Association for Australian rural nurses”) OR (MH “services for Australian rural and remote allied health”) 4. "craniocerebral injuries" OR "craniocerebral injury" OR "craniocerebral trauma" OR "craniocerebral traumas" OR "crushing skull injuries" OR "crushing skull injury" OR "forehead trauma*" OR "frontal region trauma*" OR "head injur*" OR "head trauma*" OR "occipital region trauma*" OR "occipital trauma*" OR "parietal region trauma*" OR “brain trauma*” OR “brain injur*” OR “brain laceration*” OR “epidural hemorrhage*” OR “epidural hematoma*” OR “subdural hemorrhage*” OR “subdural hematoma*” OR “intracranial hemorrhage*” OR “intracranial hematoma*” OR “brain injur*” OR “epidural haemorrhage*” or “epidural haematoma*” OR “subdural haemorrhage*” OR “subdural haematoma*” OR “intracranial haemorrhage*” OR “intracranial haematoma*” OR “intracranial hematoma*” 5. rural* OR remote* 6. 1 OR 4 7. 2 OR 5 8. 3 OR 5 9. 6 AND 7 AND 8 |
| Ovid Emcare | 1. exp craniotomy/ 2. craniocerebral trauma.mp or exp craniocerebral trauma 3. exp rural health/ or exp rural population/ or ex[ hospitals, rural/ or exp rural nursing/ or exp rural health services/ or rural.mp 4. ("craniocerebral injuries" OR "craniocerebral injury" OR "craniocerebral trauma" OR "craniocerebral traumas" OR "crushing skull injuries" OR "crushing skull injury" OR "forehead trauma*" OR "frontal region trauma*" OR "head injur*" OR "head trauma*" OR "occipital region trauma*" OR "occipital trauma*" OR "parietal region trauma*" OR “brain trauma*” OR “brain injur*” OR “brain laceration*” OR “epidural hemorrhage*” OR “epidural hematoma*” OR “subdural hemorrhage*” OR “subdural hematoma*” OR “intracranial hemorrhage*” OR “intracranial hematoma*” OR “brain injur*” OR “epidural haemorrhage*” or “epidural haematoma*” OR “subdural haemorrhage*” OR “subdural haematoma*” OR “intracranial haemorrhage*” OR “intracranial haematoma*” OR “intracranial hematoma*”) .m.p 5. (“craniectom*” OR “craniotom*” OR "burr hole*" OR “trephin*” OR “trepan*” OR “Brain surg*”).m.p. 6. (rural* OR remote*).mp [mp=title, book title, abstract, original title, name of substance word, subject heading word, organism supplementary concept word, protocol summary concept word, rare disease supplementary concept word, unique identifier, synonyms, population supplementary concept, anatomy supplementary concept word] 7. 3 OR 5 8. 2 OR 6 9. 1 OR 4 10. 7 AND 8 AND 9 |
| Ovid Medline | 1. exp craniocerebral trauma/ 2. exp craniotomy/ 3. exp rural health/ or exp rural population/ or exp hospitals, rural/ or exp rural nursing/ or exp rural health services/ or rurap.mp. 4. ("craniocerebral injuries" OR "craniocerebral injury" OR "craniocerebral trauma" OR "craniocerebral traumas" OR "crushing skull injuries" OR "crushing skull injury" OR "forehead trauma*" OR "frontal region trauma*" OR "head injur*" OR "head trauma*" OR "occipital region trauma*" OR "occipital trauma*" OR "parietal region trauma*" OR “brain trauma*” OR “brain injur*” OR “brain laceration*” OR “epidural hemorrhage*” OR “epidural hematoma*” OR “subdural hemorrhage*” OR “subdural hematoma*” OR “intracranial hemorrhage*” OR “intracranial hematoma*” OR “brain injur*” OR “epidural haemorrhage*” or “epidural haematoma*” OR “subdural haemorrhage*” OR “subdural haematoma*” OR “intracranial haemorrhage*” OR “intracranial haematoma*” OR “intracranial hematoma*”) .m.p 5. (“craniectom*” OR “craniotom*” OR "burr hole*" OR “trephin*” OR “trepan*” OR “Brain surg*”).m.p. 6. (rural* OR remote*).mp [mp=title, book title, abstract, original title, name of substance word, subject heading word, organism supplementary concept word, protocol summary concept word, rare disease supplementary concept word, unique identifier, synonyms, population supplementary concept, anatomy supplementary concept word] 7. 1 OR 4 8. 2 OR 5 9. 3 OR 6 10. 7 AND 8 AND 9 |
| Scopus | Article title, abstract, keywords: ("craniocerebral injuries" OR "craniocerebral injury" OR "craniocerebral trauma" OR "craniocerebral traumas" OR "crushing skull injuries" OR "crushing skull injury" OR "forehead trauma*" OR "frontal region trauma*" OR "head injur*" OR "head trauma*" OR "occipital region trauma*" OR "occipital trauma*" OR "parietal region trauma*" OR “brain trauma*” OR “brain injur*” OR “brain laceration*” OR “epidural hemorrhage*” OR “epidural hematoma*” OR “subdural hemorrhage*” OR “subdural hematoma*” OR “intracranial hemorrhage*” OR “intracranial hematoma*” OR “brain injur*” OR “epidural haemorrhage*” or “epidural haematoma*” OR “subdural haemorrhage*” OR “subdural haematoma*” OR “intracranial haemorrhage*” OR “intracranial haematoma*” OR “intracranial hematoma*” OR “neurotrauma”)  AND (“craniectom*” OR “craniotom*” OR "burr hole*" OR “trephin*” OR “trepan*” OR “brain surg*”) AND (rural* OR remote*) |

**Appendix 3 – QATSDD criteria and scoring^10^**

| Criteria | 0 = not at all | 1 = very slightly | 2 – moderately | 3 = complete |
| --- | --- | --- | --- | --- |
| 1. Explicit theoretical framework | No mention at all | Reference to broad theoretical basis. | Reference to a specific theoretical basis | explicit statement of theoretical framework and/or constructs applied to the research |
| 1. Statement of aims / objectives in main body of report | No mention at all | General reference to aim/objective at some point in the report including abstract. | Reference to broad aims/objectives in main body of report. | Explicit statement of aims/objectives in main body of report. |
| 1. Clear description of research setting | No mention at all | General description of research area and background, e.g. ‘in primary care’. | General description of research problem in the target population, e.g. ‘among GPs in primary care’. | Specific description of the research problem and target population in the context of the study, e.g. nurses and doctors from GP practices in the east midlands. |
| 1. Evidence of sample size considered in terms of analysis | No mention at all | Basic explanation for choice of sample size. Evidence that size of the sample has been considered in study design. | Evidence of consideration of sample size in terms of saturation/information redundancy or to fit generic analytical requirements. | Explicit statement of data being gathered until information redundancy/saturation was reached or to f it exact calculations for analytical requirements. |
| 1. Representative sample of target group of a reasonable size | No statement of target group. | Sample is limited but represents some of the target group or representative but very small. | Sample is somewhat diverse but not entirely representative, e.g. inclusive of all age groups, experience but only one workplace. Requires discussion of target population to determine what sample is required to be representative. | Sample includes individuals to represent a cross section of the target population, considering factors such as experience, age and workplace. |
| 1. Description of procedure for data collection | No mention at all | Very basic and brief outline of data collection procedure, e.g. ‘using a questionnaire distributed to staff’ | States each stage of data collection procedure but with limited detail, or states some stages in details but omits others. | Detailed description of each stage of the data collection procedure, including when, where and how data were gathered |
| 1. Rationale for choice of data collection tool(s) | No mention at all | Very limited explanation for choice of data collection tool(s). | Basic explanation of rationale for choice of data collection tool(s), e.g. based on use in a prior similar study. | Detailed explanation of rationale for choice of data collection tool(s), e.g. relevance to the study aims and assessments of tool quality either statistically, e.g. for reliability & validity, or relevant qualitative assessment. |
| 1. Detailed recruitment data | No mention at all | Minimal recruitment data, e.g. no. of questionnaire sent and no. returned. | Some recruitment information but not complete account of the recruitment process, e.g. recruitment figures but no information on strategy used. | Complete data regarding no. approached, no. recruited, attrition data where relevant, method of recruitment. |
| 1. Statistical assessment of reliability and validity of measurement tool(s) (Quantitative only) | No mention at all | Reliability and validity of measurement tool(s) discussed, but not statistically assessed. | Some attempt to assess reliability and validity of measurement tool(s) but insufficient, e.g. attempt to establish test–retest reliability is unsuccessful but no action is taken. | Suitable and thorough statistical assessment of reliability and validity of measurement tool(s) with reference to the quality of evidence as a result of the measures used. |
| 1. Fit between stated research question and method of data collection (Quantitative) | No research question stated. | Method of data collection can only address some aspects of the research question. | Method of data collection can address the research question but there is a more suitable alternative that could have been used or used in addition. | Method of data collection selected is the most suitable approach to attempt answer the research question |
| 1. Fit between stated research question and format and content of data collection tool e.g. interview schedule (Qualitative) | No research question stated. | Structure and/or content only suitable to address the research question in some aspects or superficially. | Structure & content allows for data to be gathered broadly addressing the stated research question(s) but could benefit from greater detail | Structure & content allows for detailed data to be gathered around all relevant issues required to address the stated research question(s). |
| 1. Fit between research question and method of analysis | No mention at all | Method of analysis can only address the research question basically or broadly. | Method of analysis can address the research question but there is a more suitable alternative that could have been used or used in addition to offer greater detail | Method of analysis selected is the most suitable approach to attempt answer the research question in detail, e.g. for qualitative IPA preferable for experiences vs. content analysis to elicit frequency of occurrence of events, etc. |
| 1. Good justification for analytical method selected | No mention at all | Basic explanation for choice of analytical method | Fairly detailed explanation of choice of analytical method. | Detailed explanation for choice of analytical method based on nature of research question(s). |
| 1. Assessment of reliability of analytical process (Qualitative only) | No mention at all | More than one researcher involved in the analytical process but no further reliability assessment. | Limited attempt to assess reliability, e.g. reliance on one method. | Use of a range of methods to assess reliability, e.g. triangulation, multiple researchers, varying research backgrounds. |
| 1. Evidence of user involvement in design | No mention at all | Use of pilot study but no involvement in planning stages of study design. | Pilot study with feedback from users informing changes to the design. | Explicit consultation with steering group or statement or formal consultation with users in planning of study design |
| 1. Strengths and limitations critically discussed | No mention at all | Very limited mention of strengths and limitations with omissions of many key issues. | Discussion of some of the key strengths and weaknesses of the study but not complete | Discussion of strengths and limitations of all aspects of study including design, measures, procedure, sample & analysis |
